# Impact of yoga on the central and peripheral vascular function among desk-based workers: A single-centered trial study

**DOI:** 10.12688/f1000research.135239.4

**Published:** 2025-06-16

**Authors:** Poovitha Shruthi P, Koustubh Kamath, Vaishali K, Shivashankar K N, Suresh Sukumar, Sneha Ravichandran, Leena R David, Peter Hogg, Guruprasad V, Banumathe K R, Shovan Saha, Rajagopal Kadavigere

**Affiliations:** 1 Dept of Yoga, Center for Integrative Medicine and Research, Manipal Academy of Higher Education, Manipal, Karnataka, 576104, India; 2 Dept of Medical Imaging Technology , MCHP, Manipal Academy of Higher Education, Manipal, Karnataka, 576104, India; 3 Dept of Physiotherapy, Manipal College of Health Professions ManipalCHP, Manipal Academy of Higher Education, Manipal, Karnataka, 576104, India; 4 Dept of Medicine, Kasturba Medical College Manipal, Manipal Academy of Higher Education, Manipal, Karnataka, 576104, India; 5 Dept. of Medical Diagnostic Imaging, University of Sharjah, Sharjah, Sharjah, 500001, United Arab Emirates; 6 School of health and Society, University of Salford, Salford, England, UK; 7 Dept. of Occupational therapy, Manipal College of Health Professions Manipal, Manipal Academy of Higher Education, Manipal, Karnataka, 576104, India; 8 School of Rehabilitation and Medical Sciences, College of Health Sciences, University of Nizwa, Sultanate of Oman, Oman; 9 Program Director, Occupational Therapy, Manipal University College, Jalan Batu Hampar,75150, Bukit Baru, Malaysia; 10 Department of Radiodiagnosis and Imaging, Kasturba Medical College Manipal, Manipal Academy of Higher Education, Manipal, Karnataka, 576104, India

**Keywords:** Physical Activity, Vascular Function, FMD, Exercise, Sedentary lifestyle

## Abstract

**Background:**

The aim of this study was to observe and analyze vascular function in ‘prolonged sitting’, followed by a yoga asana routine and pranayama intervention. Participants in this study include those who work from desks in offices. The study required the participants to attend on three separate days at random, and they had to finish a computerized test on each day. On the first day, participants were required to complete a computer test while sitting still for four hours (with the exception of washroom breaks). The next day, they underwent a computerized test along with a pranayama intervention. Finally, on the last day, they underwent a computerized test along with a yoga asana intervention. At the start of the study and after two and four hours, we measured the diameter and velocity of the common carotid artery (CCA) and superficial femoral artery (SFA).

**Methods:**

The study was a within-subjects prospective single-center trial conducted in the Department of Radio-Diagnosis and Imaging, Kasturba Medical Hospital, Manipal, India, between September 2022 and January 2023. Participants were asked to do one of the following ‘activities’ over successive weeks: Week 1 – Prolonged sitting; Week 2 – Pranayama intervention; and Week 3 – Yoga asana intervention during prolonged sitting. The baseline and follow-up variables of pulse velocity, endothelial thickness, and shear rate were assessed for normality through a Shapiro-Wilk Test.

**Results:**

Our sample included 11 participants with moderate physical activity, five with high physical activity and one with low physical activity. Yoga asana intervention comprised participants sitting continuously for four hours, with a yoga asana intervention being provided every hour, lasting for 10 minutes.

**Conclusions:**

Yoga asana improves vascular functions in prolonged sitting conditions. This routine can promote the concept of interrupted sitting and ways to reduce it with efficient yoga asana practice without changing the work culture and provide better physical relief.

**Trial registration:**

**Clinical Trials Registry – India (** CTRI/2022/09/045628), date of registration: 19/09/2022( CTRI/2022/9/045628) https://ctri.nic.in/Clinicaltrials/main1.php?EncHid=16349.27799,

**Abbreviations**
BMI: Body mass index
CCA: Common carotid artery
FMD: Flow mediate dilation
IEC: Institutional ethics committee
IPAQU: International Physical Activity Questionnaire
KH: Kasturba Hospital
KMC: Kasturba Medical College
LPA: Lower physical activity
MCA: Middle cerebral artery
SFA: Superficial femoral artery
WHO: World Health Organization

## Introduction

For a growing proportion of the world’s population, the impact of the digital working era combined with a tendency to adopt an overall sedentary lifestyle has resulted in people sitting for prolonged periods of time. This can lead to low energy expenditure and lower physical activity (LPA) levels. This has many adverse effects, including early onset of metabolic diseases such as hypertension, type 2 diabetes mellitus, obesity, atherosclerosis, late-life cognitive decline, loss of alertness, and a reduced reaction time. ^1^^–^^8^ For an adult, according to the World Health Organization (WHO), the average individual requires at least 150 min of exercise per week. ^9^ However, meeting this demand might not be accessible due to the pressures of modern life. As a result, extended sitting has been one strategy used to assist people in adopting a variety of postural motions, mostly in office-based working contexts. ^1^^–^^8^

A root cause of the above disorders has been linked to vascular impairment; therefore, improving vascular blood flow becomes essential in minimizing the potential of developing adverse effects in later life. ^10^ Blood vessels that are particularly affected include the superficial femoral artery (SFA) and common carotid artery (CCA). ^11^ The seated position has a marked and negative impact on SFA blood flow, being confounded by a sedentary lifestyle and working at computers. SFA flow reduction can cause damage and acute distress ^12^; similarly, CCA flow reduction can also have detrimental consequences.

Research has been published to investigate ways to reduce prolonged sitting and to offer interventions that can improve vascular function whilst minimizing disturbance to the working environment and work productivity. ^13^^–^^16^ Additional problems of interventions within the working environment are related to space for physical activity and the cost of any exercise-related equipment. For the workplace, a need exists to identify even more productive, feasible, convenient, cost-limited and practical exercises that increase blood flow without negative impact and at an acceptable cost.

It is suggested that customized yoga asana, using the pranayama routine, can improve concentration and alertness. ^17^^–^^20^ Yoga experts suggest this can improve vascular function and blood flow. However, yoga asana’s impact on CCA and SFA blood flow is unclear. Consequently, our study aimed to observe and analyse CCA and SFA blood flow in ‘prolonged sitting’, followed by a yoga asana routine and pranayama intervention.

## Methods

The study was a within-subjects prospective single-centre trial conducted in the Department of Radio-Diagnosis and Imaging, Kasturba Medical Hospital, Manipal, India, between September 2022 to January 2023. After approval from the Institutional Ethics Committee, Kasturba Medical College (KMC) and Kasturba Hospital (KH) (IEC1:108/2022) (approved on 22 July 2022) and Clinical Trial Registry of India ( CTRI/2022/9/045628) https://ctri.nic.in/Clinicaltrials/main1.php?EncHid=16349.27799, with prior written informed consent, 17 participants (desk based workers) were recruited for the study. This included nine males (mean age 25, age range 24–28) and eight females, mean age 25 (age range 24–28). Flyers in campus stores and adverts in Manipal University Trading & Property helpline MUTC a facebook based group were used to recruit participants for the study, which was conducted at Kasturba Hospital in Manipal. The participants had no history of cardiovascular or metabolic disorders, the females were not menstruating, none had depression during the study period, and none were taking medication, which might alter vascular function. Self-reported physical activity was assessed using the International Physical Activity Questionnaire ( IPAQ). ^1^ The questionnaire assesses time (frequency and duration) spent on vigorous, moderate intensity, walking activities and sitting time in hours for the past seven days. Our sample included 11 participants with moderate physical activity, five with high physical activity and one with low physical activity; the IPAQ data is shown in Table 1.

**Table 1.** International Physical Activity Questionnaire (IPAQ) results and Body Mass Index (BMI) of the participants.

Height (cm)	Weight	Height (m)	BMI	IPAQ
167.64	60	1.674	21.411	MODERATE
172	75	1.72	25.351	HIGH
179.832	70	1.798	21.653	MODERATE
167.64	76	1.6764	27.043	HIGH
188.976	92	1.88	25.761	HIGH
189	75	1.89	20.996	MODERATE
187.9	70	1.879	19.826	MODERATE
189	75	1.89	20.996	MODERATE
167.64	50	1.674	17.842	LOW
172	60	1.72	20.281	MODERATE
167.64	50	1.67	17.928	MODERATE
161	75	1.61	28.934	HIGH
167.64	58	1.674	20.697	MODERATE
172	70	1.72	23.661	MODERATE
169	72	1.69	25.209	HIGH
170	60	1.7	20.761	MODERATE
175	75	1.75	24.489	MODERATE

### Physical activity assessment

Referring to previous population-based studies validating the IPAQ in diverse populations (Craig et al., 2003; Hagströmer et al., 2006), the questionnaire assesses time (frequency and duration) spent on vigorous, moderate-intensity, walking activities, and sitting time in hours over the past seven days. The IPAQ-short form has demonstrated acceptable reliability (Spearman’s ρ = 0.8) and criterion validity across multiple countries and populations.

### Intervention standardization and quality control

All yoga interventions were conducted by certified instructors holding RYT-500 (Registered Yoga Teacher) certification with minimum 5 years of teaching experience. Intervention protocols were standardized using detailed written scripts and video demonstrations. Each session was monitored for adherence to prescribed postures, timing, and breathing patterns. Inter-rater reliability for intervention delivery was established through dual observation of 20% of sessions (κ = 0.89).

**Figure 1.**
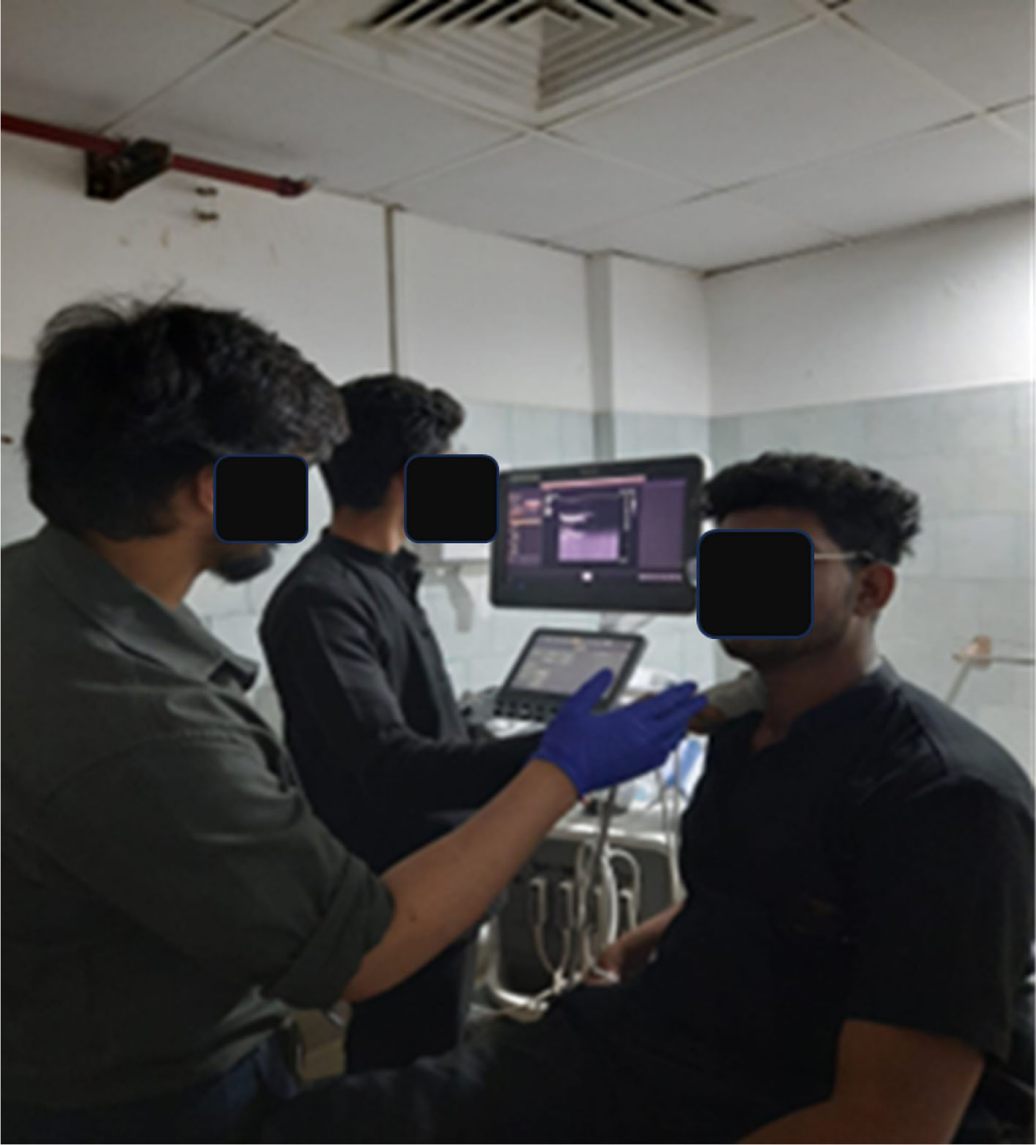
CCA measurements being taken using ultrasound.

Over three weeks, participants were requested to do one of the following ‘activities’ over successive weeks: Week 1 – prolonged sitting; Week 2 – pranayama intervention during prolonged sitting; and Week 3 – yoga asana intervention during prolonged sitting. Vascular function was assessed for each of the three conditions including the diameter and blood velocity of the SFA and CCA were measured at the start of the study (0), two, and four hours during sitting using a Doppler ultrasound (Philips, Epiq Elite). Ultrasound machine quality assurance test results fell within manufacturer specifications. Ultrasound was performed by a competent radiologist (see Figure 1).

### Detailed intervention protocols

**Figure 2.**
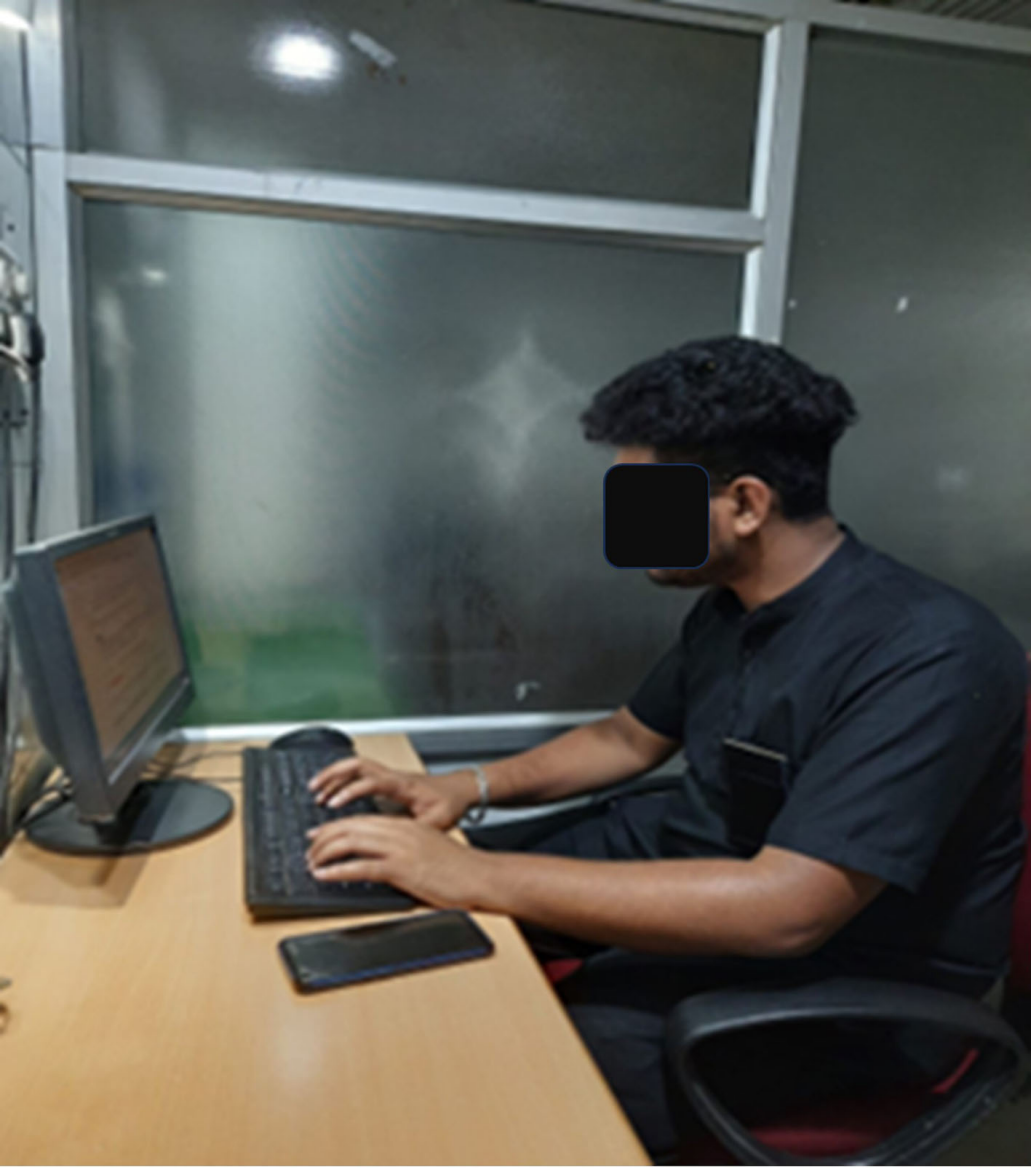
Prolonged sitting of office worker.

**Prolonged sitting:** Participants remained seated in a standardized office chair (back support 90°, feet flat on floor) for four continuous hours. Brief bathroom breaks (≤2 minutes) were permitted when necessary, but participants were instructed to minimize movement and maintain the seated position throughout the session ( Figure 2).

**Pranayama intervention:** Every hour during the 4-hour sitting period, participants performed a standardized 10-minute pranayama sequence led by a certified yoga instructor:

• Deep breathing (Dirga Pranayama): 3 minutes - slow, controlled breathing with 4-second inhalation, 2-second retention, 6-second exhalation.
• Nadisodhana Pranayama (alternate nostril breathing): 4 minutes - using right thumb and ring finger to alternate nostrils, 6-second inhalation, 6-second exhalation per nostril.
• Bhramari Pranayama (humming bee breath): 3 minutes - index fingers in ears, humming sound during exhalation.

**Figure:**
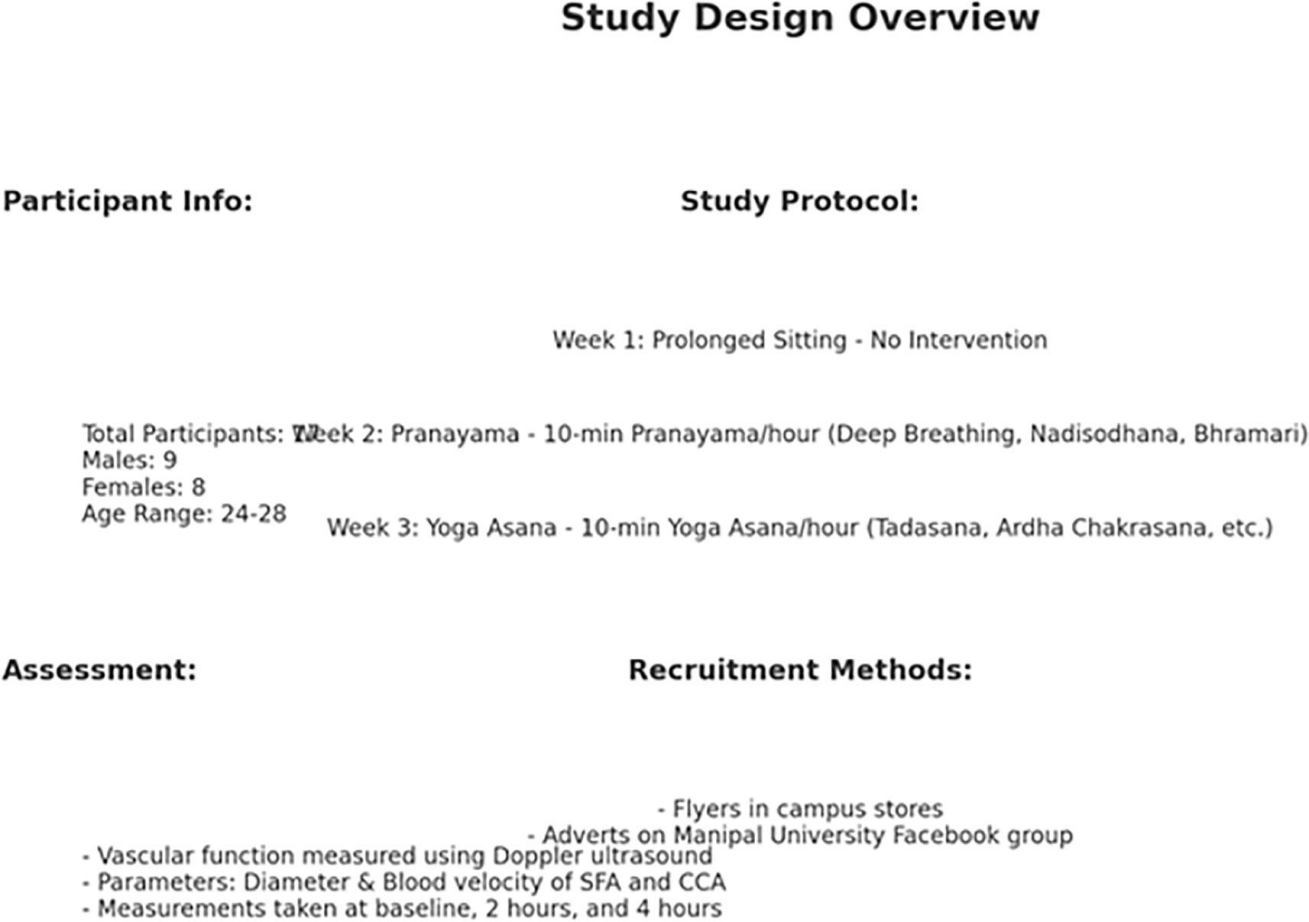
Summary of the Study Methodology.

**Yoga Asana Intervention:** Every hour during the 4-hour sitting period, participants performed a standardized 10-minute sequence:

• Tadasana (Mountain Pose): 1 minute - standing with arms overhead, deep breathing.
• Ardha Chakrasana (Half Wheel): 1.5 minutes - backward bend from standing position.
• Uttana Mandukasana (Intense Frog Pose): 2 minutes - deep forward fold with wide legs.
• Skandha Chakra Asana (Shoulder rotation): 1.5 minutes - seated shoulder rolls and stretches.
• Kati Chakrasana (Waist rotation): 2 minutes - standing waist twists.
• Prasarita Padottanasana (Wide-legged forward fold): 2 minutes - standing forward fold with wide stance.

All interventions were supervised by qualified instructors and standardized across participants to ensure consistency.

### Statistical analysis

Statistical significance was set at α = 0.05 for all analyses. Given the exploratory nature of this study with multiple outcome measures, we report both statistical significance (p < 0.05) and effect sizes to provide comprehensive interpretation. For multiple comparisons within each vascular measure (diameter, velocity, shear, stress), Bonferroni correction was applied, adjusting the significance threshold to α = 0.0125 (0.05/4) for family-wise error control. Post-hoc analyses for significant main effects used Tukey’s HSD test to control Type I error inflation. All analyses were performed using JASP statistical software version 0.16.2, with both parametric (repeated measures ANOVA) and non-parametric (Friedman’s test) approaches based on normality testing via Shapiro-Wilk test.

## Results

The study comprised 17 participants in the 25–35 age range. Nine males (mean age 25, age range 24–28) and eight females (mean age 25, age range 24–28) made up the participants. ^25^ None of the participants experienced depression throughout the study period, none had a history of cardiovascular or metabolic diseases, and none were taking any medications that would affect vascular function. Additionally, none of the female participants were menstruating. The IPAQ was used to evaluate self-reported physical activity. For the previous seven days, the questionnaire measures the amount of time (frequency and duration) spent on walking activities that are vigorous or moderately intense as well as hours spent sitting down.

### CCA diameter

**Figure 3.**
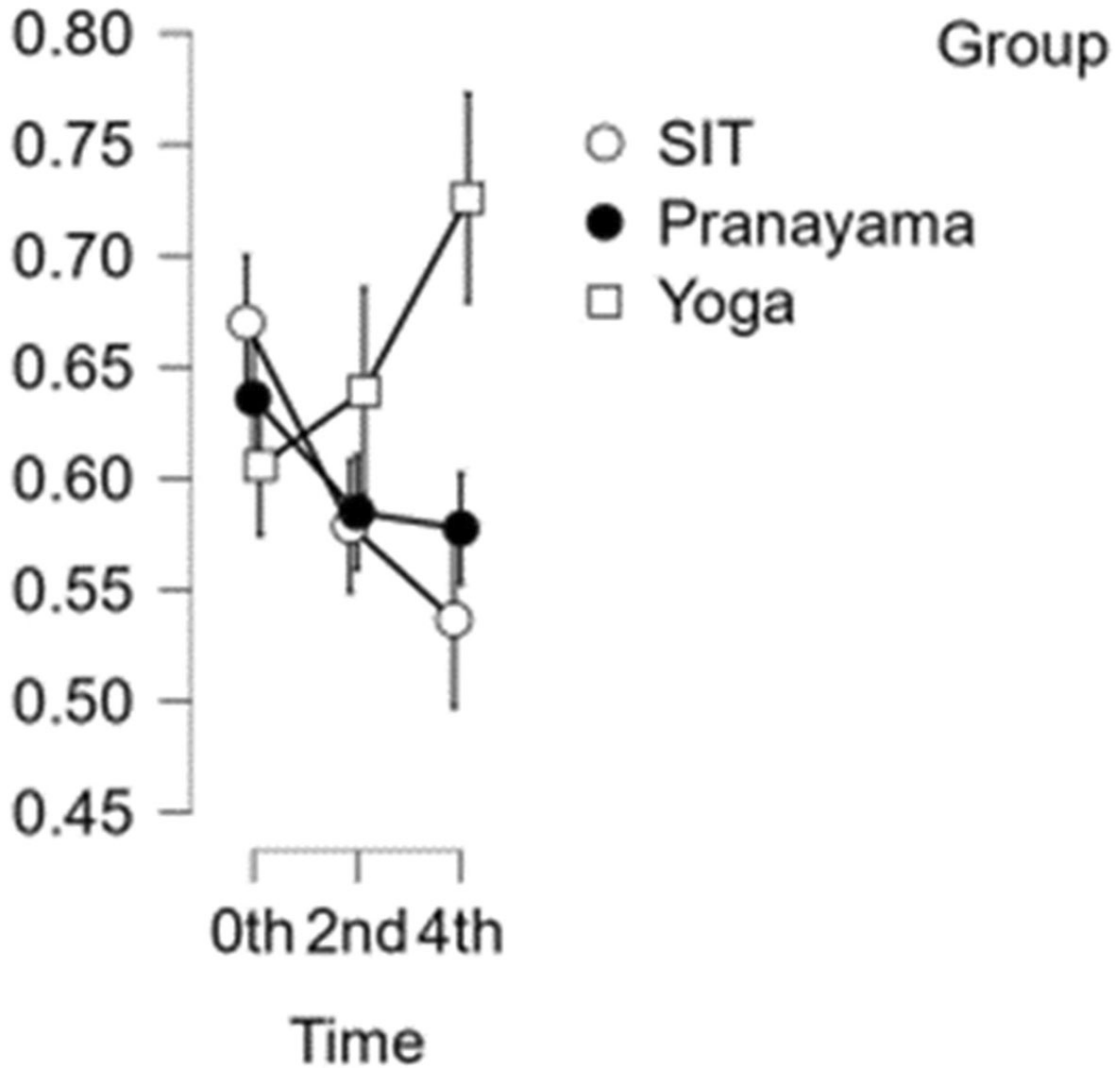
CCA diameter over the study time. (In figure X axis represents Time, Y axis represents vascular diameter (cm).)

Figure 3 illustrates how the results of the CCA reveal that the artery’s width decreases with each set of interventions. In comparison with time, the first and second hours exhibited statistical significance with a p value<0.001, whereas the third and fourth hours do not exhibit statistical significance (p value = 0.020). The yoga asana intervention demonstrated statistical significance (p = 0.014) when compared to the pranayama group and prolonged sitting. Table 2 represents the data of CCA diameter at different time points.

**Table 2.** Common carotid artery (CCA) diameter at different intervention time points.

CCA diameter			
Intervention	0 hour Mean ± SD	2 ^nd^ hour Mean ± SD	4 ^th^ hour Mean ± SD
Sitting intervention	0.670 ± 0.062	0.579 ± 0.059	0.537 ± 0.067
Pranayama intervention	0.636 ± 0.077	0.585 ± 0.059	0.577 ± 0.054
Yoga asana intervention	0.606 ± 0.067	0.639 ± 0.115	0.726 ± 0.111

In addition, the yoga asana intervention demonstrated statistical significance when compared to the pranayama intervention and prolonged sitting (p value = 0.017).

### CCA velocity

**Figure 4.**
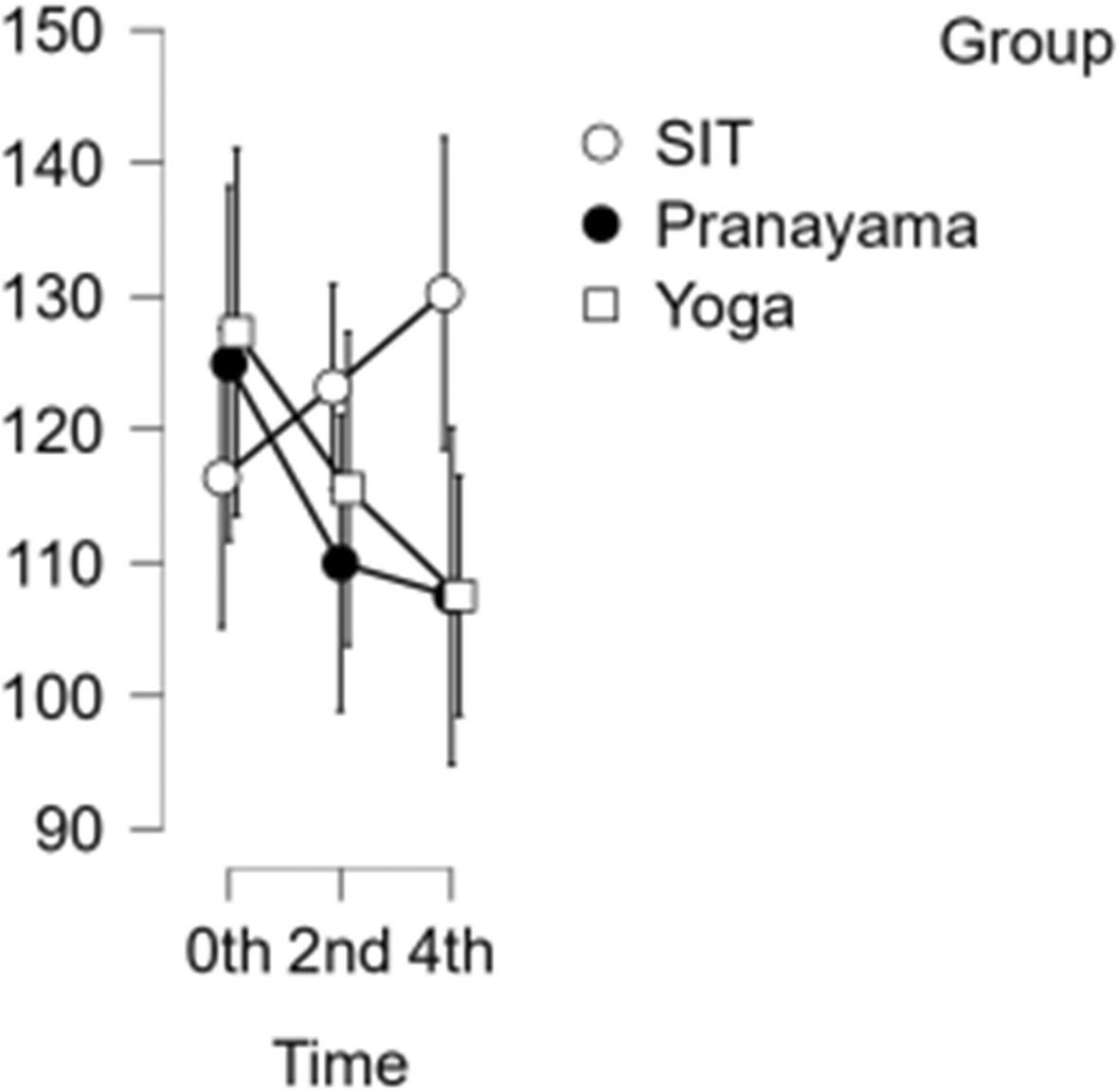
CCA Velocity in different time points. (In figure X axis represents Time, Y axis represents velocity (m/s).)

In the CCA, a reduction in velocity was observed after the pranayama and yoga asana intervention. However, when it comes to prolonged sitting, there was a decrease in velocity at the start of the study and at two hours, followed by an increase in velocity during the final hour as shown in Figure 4. With CCA velocity, neither time nor blood velocity were statistically significant. Table 3 represents the data of CCA velocity at different time points.

**Table 3.** Common carotid artery (CCA) blood velocity at different intervention time points.

CCA blood velocity			
Intervention	0 hour Mean ± SD	2 ^nd^ hour Mean ± SD	4 ^th^ hour Mean ± SD
Sitting intervention	116.365 ± 19.713	123.176 ± 11.485	130.206 ± 21.310
Pranayama intervention	124.941 ± 13.797	109.953 ± 25.810	107.482 ± 25.806
Yoga asana intervention	127.294 ± 28.458	115.524 ± 25.495	107.506 ± 17.020

### SFA diameter

**Figure 5.**
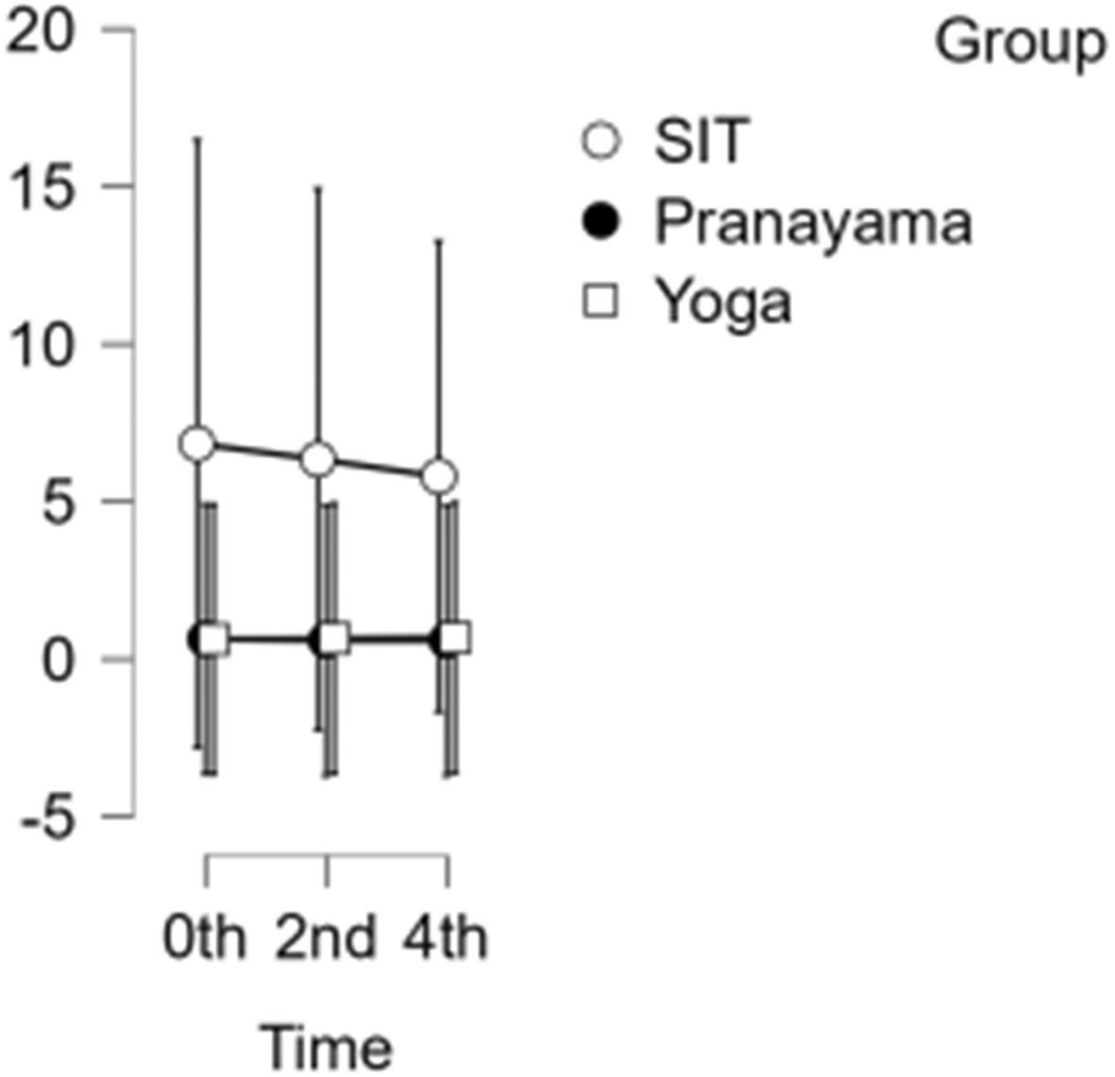
SFA diameter at different intervention time points. (In figure X axis represents Time, Y axis represents diameter in cm.)

The diameter of the SFA decreased each hour during prolonged sitting and pranayama intervention. However, during the yoga asana intervention, there was a reduction in diameter at the beginning of the study and at 2 hours, but an increase in diameter was observed in the 4 ^th^ hour, shown in Figure 5. Neither time nor group showed any significance. Hence the group has not demonstrated any substantial response to any intervention. Tables 4 & 5 represents the data of SFA diameter and velocity at different time points respectively.

**Table 4.** Superficial femoral artery (SFA) diameter at different intervention time points.

SFA Diameter			
Intervention	0 hour Mean ± SD	2 ^nd^ hour Mean ± SD	4 ^th^ hour Mean ± SD
Sitting intervention	6.833 ± 25.555	6.339 ±23.621	5.796 ± 21.570
Pranayama intervention	0.635 ± 0.051	0.591 ± 0.073	0.589 ± 0.072
Yoga asana intervention	0.626 ± 0.049	0.650 ± 0.058	0.677 ± 0.076

**Table 5.** Superficial femoral artery (SFA) Velocity values for different interventions.

SFA Velocity			
Intervention	0 hour Mean ± SD	2 ^nd^ hour Mean ± SD	4 ^th^ hour Mean ± SD
Sitting intervention	100.836 ± 42.410	94.120 ± 39.232	97.401 ± 46.217
Pranayama intervention	98.859 ± 25.082	88.276 ± 23.905	85.288 ± 15.356
Yoga asana intervention	110.847 ± 24.281	106.024 ± 25.739	98.224 ± 21.270

### CCA shear and stress

**Figure 6.**
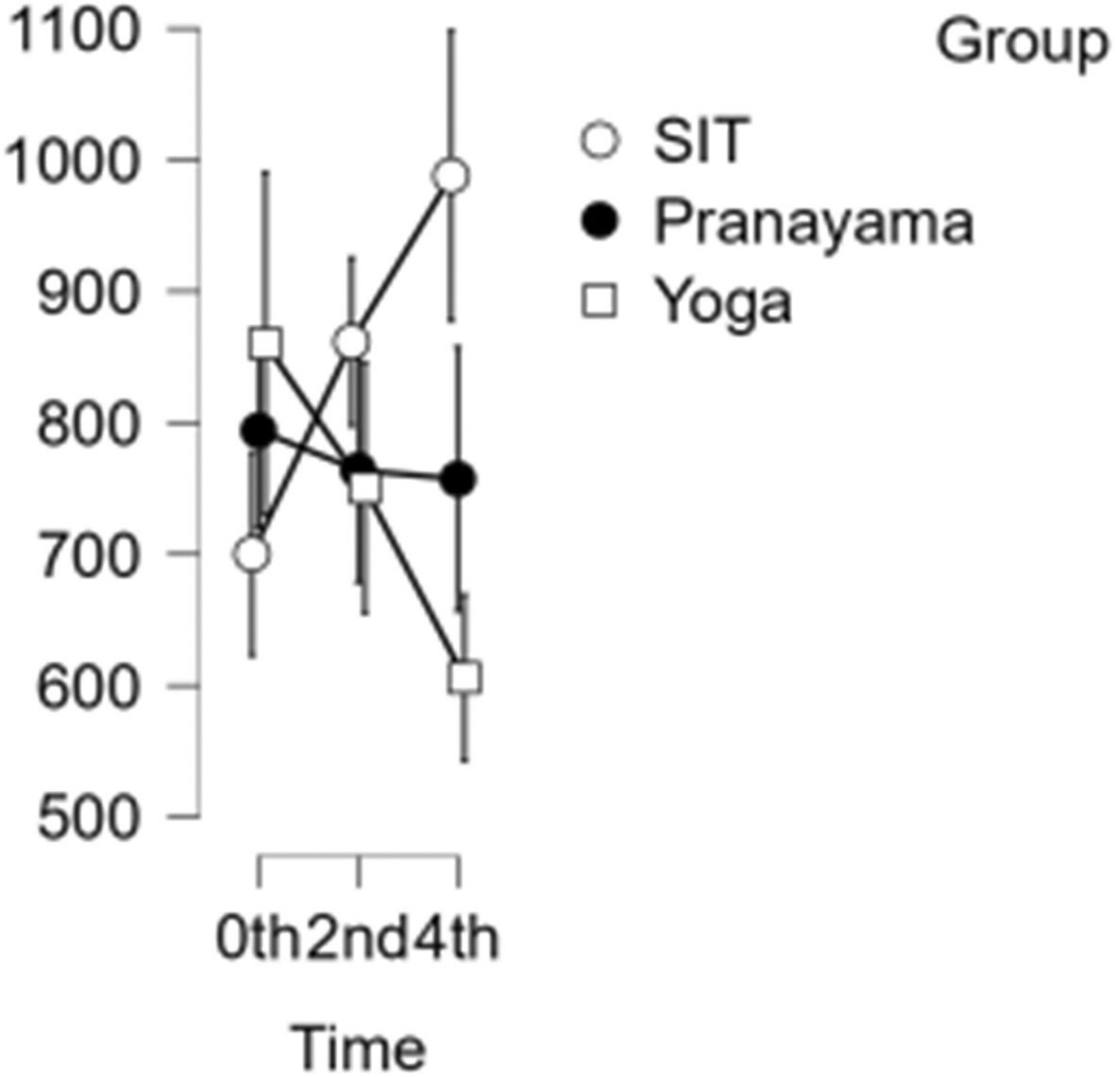
CCA shear at different intervention time points. (In figure X axis represents Time, Y axis represents velocity (m/s).)

**Figure 7.**
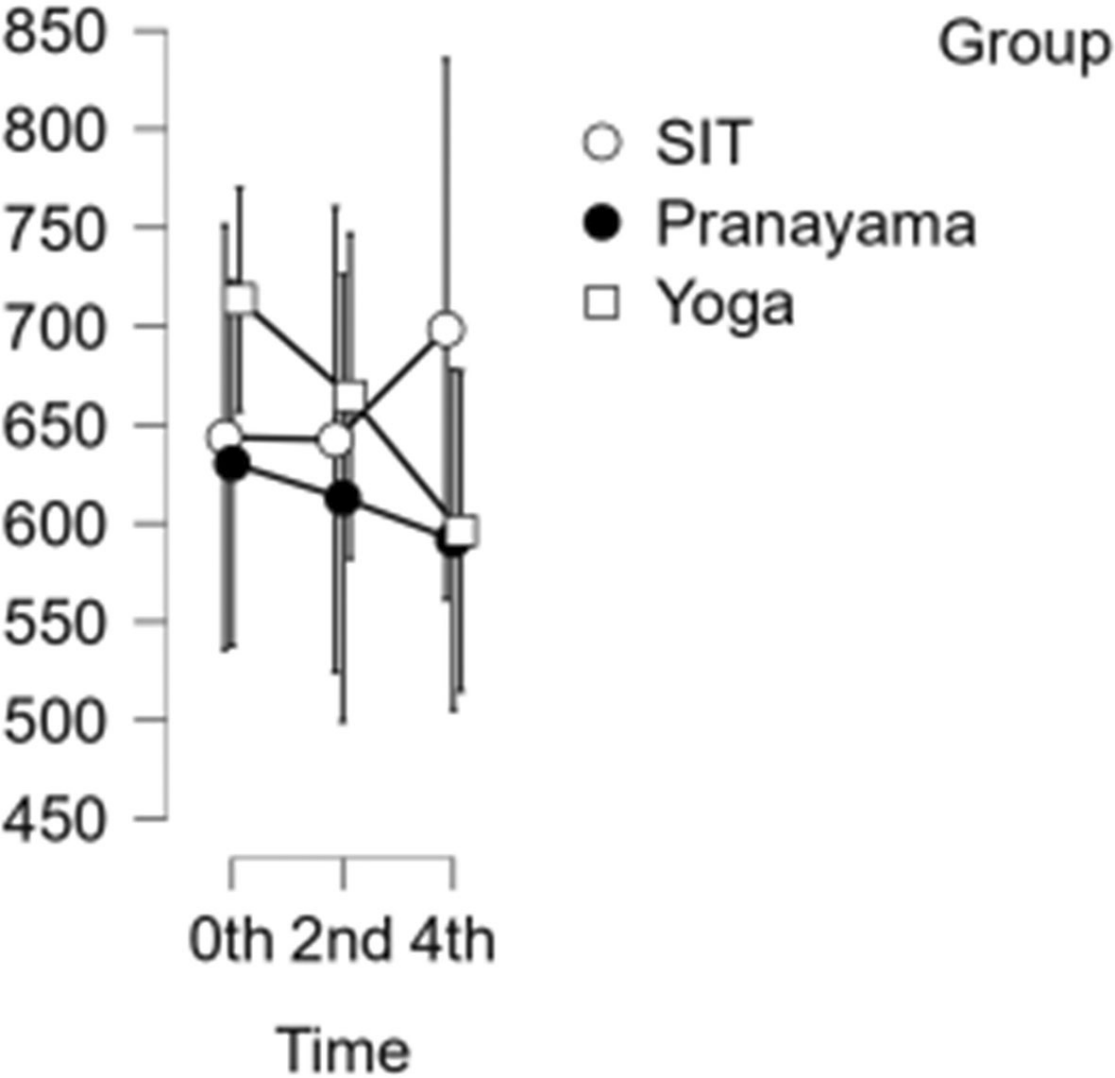
Shows CCA stress at different intervention time points. (In figure X axis represents Time, Y axis represents velocity (m/s).)

In the CCA, the shear rate increased each hour during prolonged sitting, whereas it decreased in both the pranayama and yoga asana interventions ( Figure 6). All interventions showed a reduction in stress in CCA ( Figure 7). However, in terms of shear and stress, neither the time nor the group showed any significant difference. Table 6 represents the data of CCA shear and stress at different time points.

**Table 6.** Common carotid artery (CCA) shear and stress at different intervention time points.

CCA Shear				CCA Stress		
Intervention	0 hour Mean ± SD	2 ^nd^ hour Mean ± SD	4 ^th^ hour Mean ± SD	0 hour Mean ± SD	2 ^nd^ hour Mean ± SD	4 ^th^ hour Mean ± SD
Sitting intervention	643.633 ± 235.083	642.379 ± 258.007	698.348 ± 277.646	700.154 ± 137.83	861.576 ± 127.689	987.954 ± 208.107
Pranayama intervention	630.400 ± 183.076	612.781 ± 197.767	591.701 ± 145.139	794.020 ± 201.092	763.876 ± 211.299	757.151 ± 214.785
Yoga asana intervention	713.502 ± 172.524	664.485 ± 194.142	596.131 ± 184.105	860.253 ± 268.289	750.484 ± 233.039	605.912 ± 133.750

## Discussion

Our study aimed to determine whether yoga asana affects vascular function in desk-based workers. Exercise will maintain and enhance both physical and mental health. By controlling the sympathetic nervous system and hypothalamus pituitary adrenal axis, yoga asana can improve physical and psychological health. ^21^

### Yoga asana intervention

Our study investigated acute vascular responses to single-session interventions, which differs from most existing literature examining chronic exercise effects. While studies like Ross and Thomas (2010) ^19^ examined long-term yoga effects over 3-6 months, ^19^ our findings demonstrate that even acute yoga asana interventions can produce measurable vascular improvements. This aligns with research by Thosar et al. (2015), ^23^ who similarly examined acute vascular responses during prolonged sitting interventions. ^23^ The immediate vascular benefits observed in our study suggest that yoga asanas may serve as effective ‘micro-breaks’ during prolonged occupational sitting, with potential for cumulative benefits when practiced regularly.

In a study by Pearson and Smart (2017), participants underwent aerobic exercise with an intervention duration ranging from four weeks to six months, with the frequency of sessions varying from daily to two days per week, and the time of exercise sessions ranged from 10 to 60 minutes. The study concluded that aerobic exercise training significantly improved endothelial function. ^21^ As compared to our study, the yoga asana intervention showed improvement in both carotid and superficial femoral artery diameter.

A study conducted by Cortez-Cooper examined the effect of different types of intervention on cardiovascular health. The study included three groups: a strength training group, a combination group that received both strength training and aerobic exercise, and a stretching group. The results showed that the strength training and combination groups experienced significant changes in cardiovascular health markers, including carotid artery pulse pressure. The study also found that carotid artery compliance decreased slightly in the strength training and combination groups and increased in the stretching group. However, the study found no carotid-femoral pulse wave velocity changes in the strength training and combination groups. Overall, the combination of strength training and aerobic exercise may be more effective than stretching alone in improving cardiovascular health. ^22^ When compared to our study, the yoga asana intervention gave significant changes.

### Prolonged sitting

A study by Thosar *et al*. (2015) involved 12 men participating in two randomized three-hour sitting trials. The first trial was a sitting trial where the subject was seated in one position without interruption The second trial was a sit-walk trial where the subjects walked on treadmills for five minutes at two mph for 30 minutes, 1.5-hour and 2.5-hour intervals during the sitting period. The study assessed the flow mediated dilation (FMD) of the SFA at baseline, one hour, two hours, and three hours in each trial. The results showed that there was no significant decline in the sitting trial in the FMD at each hour from baseline. The mean shear rate also showed a substantial decrease in the baseline across time. The sit-walk trial, however, showed a significant difference in the FMD and mean shear rate compared to the sit trial, indicating that walking during prolonged sitting may benefit arterial health. ^23^ As compared to our study, both CA and SFA are measured. In our study all interventions – aside from the prolonged sitting – showed a minor decrease in CA velocity over time. When the seated intervention reached the last four hours, it will rise. Compared to time, the first and second hours exhibit statistical significance with a p value of <0.001, whereas the third and fourth hours exhibit significance but do not exhibit statistical significance (p value = 0.020). There is no significant difference in SFA. But in our study, yoga asana showed effectiveness in both CA and SFA.

When people sit for lengthy periods, their CA shear increases, whereas pranayama and yoga asana interventions showed decreases in CA shear for each hour. In CA shear, both time and group will not show any significance.

Long periods of sitting have been shown to reduce stress in SFA, and yoga asana and pranayama interventions have also been shown to reduce stress over time. Both time and group do not exhibit any significant differences when compared.

The study by Carter *et al*. (2018) recruited 15 office workers (10 male). The participants sat for hours with two minutes of light intensity treadmill walking every 30 minutes and four hours sitting with an eight-minute light intensity treadmill walking break every 120 minutes. Middle cerebral artery measures were taken, and it was found that there was no significant difference in cardiorespiratory and hemodynamic measures, but a significant main effect was observed in cerebral blood flow. Post hoc analysis revealed a greater change in middle cerebral artery (MCA) during sitting compared to walking, with a considerable reduction in MCA observed in both the sitting and walking conditions. ^24^ Compared to our study, the diameter significantly decreases in the sitting intervention, but velocity will increase similarly in SFA diameter and velocity decreases during the sitting intervention. Compared to time, the first and second hours exhibit statistical significance with a p value of <0.001, whereas the third and fourth hours demonstrated significance but did not exhibit statistical significance (p value = 0.020). The yoga asana intervention showed significance compared to pranayama and prolonged sitting, though it didn’t reach statistical significance (p-value = 0.014). In contrast, the yoga asana intervention showed significance but not statistical significance when compared to the group in pranayama intervention and prolonged sitting (p value = 0.017). While in CA velocity, neither time nor velocity were significant.

The yoga asana intervention demonstrated improvement in SFA diameter; however, there was no difference between time and group when compared side by side. Each hour of the intervention resulted in a drop in velocity. The time points at the study’s start (0th hour) and at the 2nd hour demonstrate significance (p-value = 0.026). However, while both the 0th and 2nd hours show significance with a p-value of 0.004, they still do not reach statistical significance. The group did not demonstrate any substantial response to any intervention. Hence, it shows that yoga asana will improve vascular function.

### Mechanistic basis for vascular improvements

The observed improvements in CCA diameter following yoga asana intervention likely result from multiple physiological mechanisms. First, the combination of controlled movement and pranayama breathing activates the parasympathetic nervous system, reducing sympathetic vasoconstriction and promoting vasodilation through enhanced nitric oxide (NO) bioavailability. The rhythmic contraction and relaxation of skeletal muscles during asana practice creates a ‘muscle pump’ effect, enhancing venous return and reducing peripheral vascular resistance.

Second, the postural changes inherent in yoga asanas may counteract the gravitational pooling of blood that occurs during prolonged sitting. Studies have shown that even brief postural changes can restore endothelial function by increasing shear stress on arterial walls, stimulating NO production and improving flow-mediated dilation.

### Clinical implications and future directions

The observed acute improvements suggest that yoga-based micro-breaks could serve as practical occupational health interventions. However, several questions remain: (1) What is the optimal frequency and duration of interventions for sustained benefits? (2) How do individual factors (age, fitness level, cardiovascular risk) modify the response? (3) What are the long-term cardiovascular outcomes of regular yoga micro-break implementation?

Future research should investigate dose-response relationships, compare different types of movement interventions, and examine long-term cardiovascular outcomes in diverse occupational populations. Additionally, mechanistic studies using more sophisticated vascular assessment techniques (e.g., flow-mediated dilation, pulse wave velocity) could provide deeper insights into the physiological pathways involved.

## Conclusions

The findings indicate that acute yoga asana interventions may have positive effects on vascular function during prolonged sitting conditions. The theoretical basis for these effects include the activation of the parasympathetic nervous system through pranayama practices, which can reduce sympathetic vasoconstriction; mechanical muscle pump activation during asana practice, enhancing venous return and arterial flow; and improved nitric oxide bioavailability through movement-induced endothelial stimulation.

While these observations are based on single-session interventions, they align with established physiological principles and provide preliminary evidence for yoga’s acute vascular benefits. The immediate improvements in CCA diameter and the prevention of sitting-induced vascular impairment suggest that yoga asanas could serve as practical workplace interventions. However, these findings represent acute physiological responses, and longitudinal studies are essential to establish sustained clinical benefits and optimal intervention frequencies.

Many organizations globally are beginning to incorporate yoga-based movement breaks as occupational health strategies. This practice offers potential for immediate physical relief without disrupting work culture, though implementation should be guided by evidence from longer-term studies currently needed in this field.

## Data Availability

Harvard Dataverse: Underlying data for ‘Impact of Yoga on the central and peripheral vascular function among desk-based workers’, https://doi.org/10.7910/DVN/GLGPCZ. ^25^ Harvard Dataverse: CONSORT checklist for ‘Impact of Yoga on the central and peripheral vascular function among desk-based workers’, https://doi.org/10.7910/DVN/GLGPCZ. ^25^ Data are available under the terms of the Creative Commons Zero “No rights reserved” data waiver (CC0 1.0 Public domain dedication).
