# Supplementary material for: Impact of yoga on the central and peripheral vascular function among desk-based workers: A single-centered trial study
Source: F1000Res. 2025 Jun 16;13:277. Originally published 2024 Apr 15. [Version 4] doi: 10.12688/f1000research.135239.4 (PMC12231365; doi:10.12688/f1000research.135239.4)
Supplement: Peer review history

# Reviewer report: Reviewer response for version 4

**DOI:** 10.5256/f1000research.183521.r392429

**Published:** 2025-07-03

**Authors:** Mansoor Rahman

**Affiliations:** 1 MGM Institute of Physiotherapy, Chh. Sambhajinagar, India; 2 Paediatric Physiotherapy, MGM institute of Physiotherapy, Chh. Sambhajinagar, Maharshtra, India

Manuscript can be accepted with its current state.

Is the work clearly and accurately presented and does it cite the current literature?

No

If applicable, is the statistical analysis and its interpretation appropriate?

Yes

Are all the source data underlying the results available to ensure full reproducibility?

No

Is the study design appropriate and is the work technically sound?

Partly

Are the conclusions drawn adequately supported by the results?

Partly

Are sufficient details of methods and analysis provided to allow replication by others?

Partly

Reviewer Expertise:

Physical activity for cerebral palsy

I confirm that I have read this submission and believe that I have an appropriate level of expertise to confirm that it is of an acceptable scientific standard.

# Reviewer report: Reviewer response for version 3

**DOI:** 10.5256/f1000research.176793.r376041

**Published:** 2025-04-07

**Authors:** Yanwei You

**Affiliations:** 1 Tsinghua University, Beijing, Beijing, China

This study investigates the effects of yoga postures and pranayama interventions on the hemodynamic function of the common carotid artery (CCA) and superficial femoral artery (SFA) in sedentary settings. The research topic is of practical significance, especially against the backdrop of increasingly widespread digital working and the growing prevalence of sedentary lifestyles. The study proposes to improve vascular function through low-cost, easy-to-implement intervention methods, demonstrating both theoretical value and practical potential. The research design employs an within-subject experimental scheme, with a clear intervention process and the use of multi-timepoint and multi-indicator measurements, which enhances the credibility of the results. However, the manuscript has some issues in terms of structural expression, data interpretation, and literature support, and the following modifications are recommended:

 First, although the research design section provides some descriptions, certain information appears somewhat disorganized. Specifically, the definition of "prolonged sitting" should be more clearly defined, such as whether bathroom breaks are allowed and whether lower limb movement is completely restricted. Regarding the yoga postures and pranayama exercises, it is recommended to supplement details on their standardization, quality control during execution, and the qualifications of the instructors guiding the practices. Additionally, while the IPAQ questionnaire is mentioned to assess physical activity levels, there is a lack of reference to previous studies. The author is advised to revise this section and add relevant references: "Referring to previous population-based studies [You Y, et al., 2025 (Ref 1); You Y, et al., 2025 (Ref 2)], the questionnaire assesses the time (frequency and duration) spent on vigorous, moderate-intensity, walking activities, and sitting time in hours over the past seven days."

 Second, while the results section provides multiple data points and figures, there are issues with the unclear interpretation of statistical significance. For example, phrases such as "showing significance but not statistically significant" can lead to misunderstandings. The author is advised to strictly differentiate between "significant trends" and "statistical significance" and maintain consistency in the interpretation of charts and text. Additionally, certain p-values, such as 0.014 and 0.017, are described as "not statistically significant," which requires clarification in relation to the significance criteria set by the author. If multiple comparisons are performed, the author should explain whether corrections were made and which method was used (e.g., Bonferroni or FDR).

 Third, while the discussion section includes a rich array of literature references, many are simply listed in parallel without in-depth comparisons or explanations of the mechanisms underlying the study's findings. For example, the potential mechanisms by which yoga postures may improve vascular function, such as autonomic regulation and muscle pump effects, could be further explored. Furthermore, the observation of decreased CCA blood flow velocity but no significant changes in SFA should be reasonably explained in conjunction with existing research, rather than merely describing the phenomenon. The author is advised to strengthen the dialogue with existing research in the discussion section and propose directions for future research, such as long-term interventions or comparisons across different age groups.

 Finally, while the language expression is generally acceptable, some paragraphs are lengthy and repetitive. The author is advised to refine the language for greater conciseness and logical clarity during revisions. For example, the repetition of the same information in both the "Results" and "Discussion" sections can increase the reader's cognitive burden. Further optimization of paragraph structure is recommended to enhance overall readability.

Is the work clearly and accurately presented and does it cite the current literature?

Partly

If applicable, is the statistical analysis and its interpretation appropriate?

Yes

Are all the source data underlying the results available to ensure full reproducibility?

Partly

Is the study design appropriate and is the work technically sound?

Yes

Are the conclusions drawn adequately supported by the results?

Partly

Are sufficient details of methods and analysis provided to allow replication by others?

Partly

Reviewer Expertise:

Physical Activity, Sleep, Cognitive Function

I confirm that I have read this submission and believe that I have an appropriate level of expertise to confirm that it is of an acceptable scientific standard, however I have significant reservations, as outlined above.

## References

[1] : Dose-response relationship between leisure-time physical activity and metabolic syndrome in short sleep US adults: evidence from a nationwide investigation. *Appl Physiol Nutr Metab* .2025;50: 10.1139/apnm-2024-0347 1-10 10.1139/apnm-2024-0347 39993280

[2] : Mediation role of telomere length in the relationship between physical activity and PhenoAge: A population-based study. *Journal of Exercise Science & Fitness* .2025;23(3) : 10.1016/j.jesf.2025.03.004 149-156 10.1016/j.jesf.2025.03.004 PMC1199430440235556

# Reviewer report: Reviewer response for version 3

**DOI:** 10.5256/f1000research.176793.r371404

**Published:** 2025-03-21

**Authors:** Sudhanshu Kacker

**Affiliations:** 1 Rajasthan University of Health Sciences, Jaipur,, Rajasthan,, India

1. The manuscript's topic is novel and interesting. However, the authors must put more effort into rewriting and paraphrasing sentences
2. Remove hypothetical facts from the conclusion; *Yoga asana improves vascular functions in prolonged sitting conditions*
3. Introduction: Overall, the introduction is not influential. This portion needs to be rewritten.  with some relevant studies highlighting the gap area and adding the need for the study. Add recently published relevant studies on the effect of yoga on vascular functions

Add reference of this line, *Yoga experts suggest this can improve vascular function and blood flow*

1. Methods:

1. Specify the eligibility criteria.
2. How were individuals recruited, contacted, screened, and consented to
3. Validation of the 10-minute yoga protocol,
4. Criteria for sample size calculation
5.  Please elaborate on all details, including consort flow chart, adherence, fidelity, and safety of yoga, supervision, and compliance; need details of digital mode
6. What are the strategies for sustaining participants in the study?
7. Details of sociodemographic variables missing The author should add  details of education level, socioeconomic status, residence, and knowledge of yoga in sociodemographic variables
8. Statistical analysis is not appropriate. Details of secondary outcomes were missing
9. The Summary chart is not appropriate to replace it with a colorful illustration chart

3. Results

1. How did the author assure this

*None of the participants experienced depression throughout the study period, none had a history of cardiovascular or metabolic diseases, and none were taking any medications that would affect vascular function. Additionally, none of the female participants were menstruating*.

 4. Discussion

 The discussion is poorly written. Please discuss your(authors) results clearly and link to the earlier findings. The author rewrites the discussion and compares it with studies based on vascular functions and yoga. The information presented in the discussion section doesn’t link to each other.

 Remove hypothetical vague words.

*By controlling the sympathetic nervous system and hypothalamus pituitary adrenal axis, yoga asana can improve physical and psychological health*

 5. Conclusion

 The author did not mention the limitations of the study.

  The conclusion section should be revised. All references are checked according to journal guidelines.

Is the work clearly and accurately presented and does it cite the current literature?

Partly

If applicable, is the statistical analysis and its interpretation appropriate?

Partly

Are all the source data underlying the results available to ensure full reproducibility?

Partly

Is the study design appropriate and is the work technically sound?

Partly

Are the conclusions drawn adequately supported by the results?

Partly

Are sufficient details of methods and analysis provided to allow replication by others?

Yes

Reviewer Expertise:

YOGA,DIABETES.,HIGH RISK CVD,VO2 MAX,PHYSICAL ACTIVITY

I confirm that I have read this submission and believe that I have an appropriate level of expertise to confirm that it is of an acceptable scientific standard, however I have significant reservations, as outlined above.

# Response

**Published:** 2025-04-06

**Authors:** Rajagopal Kadavigere

**Affiliations:** Kasturba Medical College, Manipal, India

We sincerely appreciate the reviewer’s thoughtful feedback, which has significantly strengthened our manuscript. Below is a point-by-point response to the suggestions:  

 1. **Introduction**  

    - **Revision**: Rewrote theintroduction to better highlight the study’s novelty, citing recent literature on yoga’s vascular benefits. Added a clear gap statement and the need for the study.  

    - **Reference for yoga experts’ claim**: support the line about yoga improving vascular function.  

 2. **Methods**  

    - **Eligibility criteria**: Explicitly stated inclusion/exclusion criteria (e.g., no cardiovascular disorders, medication-free, non-menstruating females).  

    - **Recruitment**: Detailed the process (flyers, digital ads), screening (IPAQ, medical history), and consent (written).  

    - **Yoga protocol**: Cited validation for the 10-minute routine (Reference 31) and described supervision/fidelity checks (video recordings, logs).  

    - **Sample size**: Added power calculation (G*Power, effect size 0.5).  

    - **Sociodemographics**: Included education, socioeconomic status, residence, and prior yoga experience.  

    - **Statistical analysis**: Clarified secondary outcomes (shear/stress) and tests used (ANOVA/Friedman’s).  

    - **Figure**: Replaced the summary chart with a CONSORT flow diagram.  

 3. **Results**  

    - **Participant assurance**: Clarified screening methods (PHQ-9 for depression, baseline medical checks).  

    - **Tables**: Highlighted key findings (e.g., yoga’s significant CCA improvement, *p* < 0.001).  

 4. **Discussion**  

    - **Hypothetical claims**: Deleted unsupported statements (e.g., sympathetic nervous system effects).  

 5. **Conclusion**  

    - **Revised**: Removed speculative language and added study limitations (small sample size, single-center design).  

 **Additional Changes**:  

 - Improved paraphrasing throughout to enhance clarity.  

 - Added a "Limitations" subsection to the discussion.  

 We believe these revisions address all concerns and hope the manuscript now meets the journal’s standards. Thank you for your time and valuable input.

# Reviewer report: Reviewer response for version 2

**DOI:** 10.5256/f1000research.165443.r351086

**Published:** 2024-12-23

**Authors:** Mansoor Rahman

**Affiliations:** 1 MGM Institute of Physiotherapy, Chh. Sambhajinagar, India; 2 Paediatric Physiotherapy, MGM institute of Physiotherapy, Chh. Sambhajinagar, Maharshtra, India

Is the work clearly and accurately presented and does it cite the current literature? - There is mismatch in discussion references with duration of this study intervention with the existing evidences chosen to agree the study findings.

 Are all the source data underlying the results available to ensure full reproducibility? - Intervention procedures and descriptions were not mentioned in detail.

 Are the conclusions drawn adequately supported by the results? - With single session to conclude requires a strong evidences with theoretical background.

Is the work clearly and accurately presented and does it cite the current literature?

No

If applicable, is the statistical analysis and its interpretation appropriate?

Yes

Are all the source data underlying the results available to ensure full reproducibility?

No

Is the study design appropriate and is the work technically sound?

Partly

Are the conclusions drawn adequately supported by the results?

Partly

Are sufficient details of methods and analysis provided to allow replication by others?

Partly

Reviewer Expertise:

Physical Activity, Cerebral Palsy. Adapted Physical education, traditional Indian games for Physical activity for individuals with neurodevelopmental disabilities.

I confirm that I have read this submission and believe that I have an appropriate level of expertise to confirm that it is of an acceptable scientific standard, however I have significant reservations, as outlined above.
